# Sleeping Worries Away or Worrying Away Sleep? Physiological Evidence on Sleep-Emotion Interactions

**DOI:** 10.1371/journal.pone.0062480

**Published:** 2013-05-01

**Authors:** Lucia M. Talamini, Laura F. Bringmann, Marieke de Boer, Winni F. Hofman

**Affiliations:** 1 Department of Psychology, University of Amsterdam, Amsterdam, The Netherlands; 2 Cognitive Science Center Amsterdam, University of Amsterdam, Amsterdam, The Netherlands; Imperial College London, United Kingdom

## Abstract

Recent findings suggest that sleep might serve a role in emotional coping. However, most findings are based on subjective reports of sleep quality, while the relation with underlying sleep physiology is still largely unknown. In this study, the impact of an emotionally distressing experience on the EEG correlates of sleep was assessed. In addition, the association between sleep physiological parameters and the extent of emotional attenuation over sleep was determined. The experimental set up involved presentation of an emotionally neutral or distressing film fragment in the evening, followed by polysomnographic registration of undisturbed, whole-night sleep and assessment of emotional reactivity to film cues on the next evening. We found that emotional distress induced mild sleep deterioration, but also an increase in the proportion of slow wave sleep (SWS) and altered patterning of rapid eye movement (REM) sleep. Indeed, while REM sleep occurrence normally increases over the course of the night, emotional distress flattened this distribution and correlated with an increased number of REM periods. While sleep deterioration was negatively associated to emotional attenuation over sleep, the SWS response was positively related to such attenuation and may form part of a compensatory response to the stressor. Interestingly, trait-like SWS characteristics also correlated positively with the extent of emotion attenuation over sleep. The combined results provide strong evidence for an intimate reciprocal relation between sleep physiology and emotional processing. Moreover, individual differences in subjects' emotional and sleep responses suggest there may be a coupling of certain emotion and sleep traits into distinct emotional sleep types.

## Introduction

Accumulating evidence suggests that emotions and sleep may be intimately related. For example, the majority of dream reports have an emotional content, with negative emotions, such as fear and anxiety, being more frequent than positive ones [1]. These emotions, as well as the conceptual content of dreams, are related to presleep experience [2].

The relation between sleep and emotional processes appears to be reciprocal. Indeed, chronic stress and traumatizing life events are known to negatively affect sleep, resulting in chronic sleep problems [3], [4]. Chronic sleep loss, in return, affects mood [5], making people emotionally labile, irritable and more sensitive to dysphoria [6], [7], [8], [9]. Such mood alterations are associated with amplified neural and autonomic responses to emotional stimuli [10], [11], [12], possibly due to reduced inhibition by higher onto lower order affective brain areas [10], [13].

An interaction between sleep and emotional processing may also play a role in affective disorders, most of which are associated with sleep disturbance. In the past these sleep problems were considered a secondary symptom of the affective disorder. However, recent studies suggest that this view may be too simple. In some cases at least, insomnia appears to contribute to the genesis and maintenance of depression [14], [15], [16]. Furthermore, there is some evidence for a common cause underlying depression and insomnia [17]. In a similar way, recent research provides some support for the importance of sleep disturbances in the development and perpetuation of post-traumatic stress disorder (PTSD) [4], [18].

A final link between sleep and emotion comes from studies suggesting that memories, including emotional ones, are reprocessed and reorganized during sleep. In support of this notion, studies in rodents have shown ‘replay’ of task-related neuronal firing patterns during subsequent sleep [19]. This replay is related to memory acquisition [20]. Similarly, brain imaging in humans has revealed sleep-related reactivation of task-relevant cerebral areas [21] and electrophysiological sleep parameters have been correlated with learning [22], [23] and cross-sleep memory retention [24]. Moreover, sleep has been shown to benefit memory performance [25], [26], [27], and more specifically emotional memory performance [28], [29], [30], [31], in several ways. The combined observations across these various lines of research suggest that emotional memories may be reprocessed during sleep, possibly as part of some coping mechanism [2]. During such reprocessing, important memory content may be enhanced, while associated emotions may be depotentiated or uncoupled [32], . Despite the broad interest in the relation between emotional processing and sleep, and its obvious importance with regard to affective and sleep pathology, studies assessing how emotionality may influence sleep architecture are scarce. Sleep architecture regards the occurrence and temporal pattering of sleep stages, including light sleep, slow wave sleep (SWS) and rapid eye movement (REM) sleep. These stages, each with its own neurophysiological, neurochemical and general physiological fingerprint, may contribute differentially to emotional memory processing. Indeed, some observations especially implicate REM sleep herein [34], [35], [36]. However, the interaction between emotion and sleep architecture, and the putative functional relevance of different sleep stages in this interaction, is as yet poorly understood.

As a step towards understanding this interaction, the current study aims to characterize the sleep-architectural response to an induced, aversive, emotional experience in healthy subjects. A few previous studies, mainly from around the nineteen seventies, have investigated effects of psychosocial stressors on sleep and dreaming, using aversive film fragments [37], [38], [39], [40] or perfunctory treatment [41] to induce a negative mood state. However, these studies generally considered a limited sample size and restricted aspects of sleep architecture. Also, the impact of the chosen stressor was not always quantified and most of these studies were focused on dream recall, which entails repeated sleep interruptions. These circumstances may have contributed to the inconsistency of results regarding stressor effects on sleep architecture.

We, therefore, performed a comprehensive laboratory experiment on a sizeable population sample (N = 32) and compared the effects of an emotionally distressing film fragment and an emotionally neutral one on sleep architecture in the ensuing night. The negative emotional stimulus triggered a profound emotional response that could be reactivated, in dampened form, following sleep. In contrast to previous studies, a broad array of sleep architectural parameters was considered, including the proportional occurrence of all sleep stages and their distribution over the night. While the study's primary aim was to assess the effects of emotional experience on sleep architecture, the inverse relation, between sleep architecture and emotional attenuation over sleep, was also investigated.

## Materials and Methods

### Ethics statement

All subjects provided written informed consent to participate in this study and the University of Amsterdam Ethics Committee approved the experiment.

### Participants

Thirty-two healthy subjects (23F/9M; mean age = 20.09; SD = 1.5) participated in the experiment. They were good sleepers, as assessed by a health questionnaire, had no prior history of neurological, psychiatric or sleep disorders, had a habitual sleep pattern of at least 7 hours sleep per night, between 11 PM and 9 AM, and had not previously seen the films from which the stimuli were derived.

Participants were asked to refrain from the use of alcohol or other drugs for two days before and during each experimental session. During the experimental sessions, participants were also asked to abstain from caffeine intake after 6 PM. Subjects maintained normal bedtimes (bedtime between 11:00 PM and 1:00 AM and waking up between 7 and 10:00 AM) and did not take naps during five days before each experimental session, as verified by sleep logs and, during the last 24 hours, with actigraphy (Actiwatch, Cambridge Neurotechnology).

### Procedure

Subjects spent two nights at the sleep laboratory, separated by at least a week. On one of the nights, sleep was preceded by watching an emotionally distressing film fragment (extract from ‘The Passion of the Christ’, Mel Gibson); on the other night, an emotionally neutral film fragment (extract from ‘March of the Penguins’, Luc Jacquet) was presented. The emotional fragment showed Jesus Christ being brutally tortured and humiliated by Roman soldiers. In the neutral fragment, Emperor penguin chicks hatched and explored their environment in Antarctic springtime. Both film fragments lasted ten minutes and were presented between 9.30 and 10.30 PM. The order of the film conditions was counterbalanced across subjects.

Subjects went to bed between 11 and 12 PM and were given the opportunity to sleep during a 9-hour lights off period. During sleep, EEG (F3, F4, C3, C4, O1, referenced to linked mastoids), EOG and EMG were acquired with a 16-channel Monet polysomnographic recording system (sampling rate: 200 Hz, high-and low-pass filter 0,03 and 70 Hz respectively, notch filter 50 Hz) and REMbrandt software (Medcare Automation BV, Amsterdam, the Netherlands). The next morning subjects rated their sleep quality on the previous night on a Dutch sleep quality scale [42] and left the laboratory. That same evening, between 8 and 9 PM, they returned to the lab and were presented with six stills from the pertaining film fragment (1 s per still, inter-still interval 9 s) to cue the memory of the original viewing.

Emotional responses to the film fragments and stills were assessed using the Dutch shortened version of the Profile of Mood States (POMS) [43]. The depression, anger and tension/anxiety scales (the latter will henceforward be referred to as tension scale) differentiated best between the emotions induced by the two film fragments in pilot studies and were used in all analyses. Subjects also rated their emotional state on a valence free, 9-point mood scale (visual analog scale), with 1 representing “very positive mood”, 5 “neutral mood” and 9 “very negative mood”. This scale will henceforward be referred to as ‘global mood scale’.

In addition to emotional state, memory for film content was tested, both after viewing the film and after viewing the stills on the next evening. This was done to check that subjects had paid attention to the film fragments, that the two fragments were encoded, on average, to a similar extent and that declarative memory for the films was still present at the time of cueing. Memory was assessed through multiple-choice questions that included three possible answers and an “I don't know” option (subjects were explicitly requested not to guess). Questions dealt with several aspects of the film fragments, such as visual and auditory information, perceptual and conceptual information, and central and peripheral aspects of the storyline. The emotional and neutral memory questionnaires were matched for the type of questions in pilot studies.

As a further control measure, subjects' sleepiness levels, before and after the film and cueing sessions, were assessed using the Dutch translation of the Stanford Sleepiness Scale [44]. Also, alertness was tested after the cueing session with a 10-minute visual psychomotor vigilance task [6].

### Data analysis

Sleep stages were scored manually according to standard criteria [45]. All subjects slept for at least 6 hours. For each sleep recording, we calculated sleep latency, total sleep time, percentages of light sleep, deep sleep (SWS) and REM sleep, latencies to stage 3 and REM sleep, the number of REM sleep episodes, REM sleep fragmentation (average number of interruptions within a REM period), the number of awakenings, minutes awake after sleep onset (WASO) and sleep efficiency. The percentages of light sleep, SWS and REM sleep were also determined for the first and second half of the night separately to assess the distribution of sleep stages over the night.

Extreme outliers, defined as data deviating more than three standard deviations from the mean, were removed. This resulted in the exclusion of one case for the analysis of the memory data and another in the analysis of the polysomnographic data. Missing values occurred only in the data of the psychomotor vigilance task. Hence, the results of this task are based on 28 instead of 32 subjects.

Comparative statistics were performed using ANOVA and t-tests for Gaussian variables; for non-Gaussian variables the Mann-Whitney U-test (independent samples) and the Wilcoxon signed-rank test (paired samples) were adopted. Correlation analyses were performed using Pearson and Spearman correlation coefficients for normally and non-normally distributed variables, respectively. For individual tests α was set at 0.05. However, where multiple correlations are tested, we also report which correlations are significant considering a family wise error rate of 0.05. To this purpose individual test α values were corrected with the Bonferroni-Holm procedure [46].

## Results

### Mood induction procedure and control variables

Pre-sleep memory for film content was similar for the emotional (*M* = 5.97, *SD* = 1.91) and neutral (*M* = 5.84, *SD* = 1.73) film fragments (*t*(30) = 0.45, *p*>0.60). Thus, any differences in sleep architecture between the film conditions cannot be interpreted as deriving from differences in memory load. Overnight memory retention was slightly higher for the emotional than the neutral film fragment, but the difference did not reach statistical significance (interaction effect between Film (emotional, neutral) and Time (pre-sleep, post-sleep): *F*(1,30) = 3.763, *p* = 0.062).

Table 1 shows baseline and response measures for the POMS scales and the global mood scale, at film viewing and at cueing with the stills. The emotional film fragment induced a strong shift towards a negative mood state, which could be reactivated, in weakened form, 24 hours later at cueing. Indeed, scores on all mood scales were significantly increased with respect to baseline after viewing the emotional film (Wilcoxon signed-rank tests and paired *t*-test, all *p*<0.05), with the most prominent increase occurring on the depression scale (158% from baseline). At cueing, baseline depression was significantly increased in the emotional condition (*M* = 8.38, *SD* = 3.20) compared to the neutral condition (*M* = 7.13, *SD* = 2.20; *z* = −2.46, *p* = 0.01), indicating either an enduring negative mood state or a reactivation of the negative mood state due to contextual cueing alone. Presentation of the stills produced an additional negative shift in global mood (*t*(31) = −3.47, *p* = 0.002), but no further significant effects on the POMS scales.

**Table 1:** Global mood and POMS scores, pre-and post-viewing the film and stills, in the emotional and neutral conditions.

	Pre-film	Post-film	Pre-cueing	Post-cueing
	*M (SD)*	*M (SD)*	*M (SD)*	*M (SD)*
**Emotional condition**				
*Global Mood scale*	4.13 (1.36)	6.31 (1.40)	3.25 (1.19)	3.97 (1.47)
POMS scales				
*Depression*	7.88 (2.71)	12.47 (5.29)	8.38 (3.20)	8.16 (3.37)
*Tension*	8.19 (3.34)	10.00 (3.85)	7.56 (3.55)	7.78 (3.30)
*Anger*	8.50 (3.88)	10.03 (4.31)	7.81 (2.43)	8.88 (4.16)
**Neutral condition**				
*Global Mood scale*	4.06 (1.16)	3.22 (1.01)	3.13 (1.21)	3.13 (1.29)
POMS scales				
*Depression*	7.81 (2.90)	7.00 (1.74)	7.13 (2.20)	7.13 (1.90)
*Tension*	8.19 (2.90)	6.41 (1.43)	7.16 (2.64)	5.72 (2.22)
*Anger*	7.38 (2.83)	7.09 (1.77)	7.81 (3.60)	7.13 (1.66)

The neutral film fragment induced a slight mood elevation, notable on all mood scales (Wilcoxon signed-rank tests and paired *t*-test, all *p*<0.05) except for the tension scale. Post-sleep cueing with the film stills did not significantly affect mood, except for a mild decrease in tension (*z* = −3.04, *p* = 0.002).

There were no differences in sleepiness or alertness between the emotional and neutral condition at any point during the experiment.

### Effects of emotional distress on sleep

Sleep architectural parameters for the emotional and neutral film night are summarized in Tables 2 and 3. The effects of emotion induction on sleep architecture were analyzed through ANOVA with factors Film (neutral, emotional) and, to assess possible effects on the distribution of sleep stages over the night, a second factor, Night-half (1^st^ half, 2^nd^ half).

**Table 2:** Sleep architectural parameters in the emotional and neutral condition for the entire night.

	ALL (N = 32)		LSQR (N = 18)		HSQR (N = 14)	
Condition	Emotional	Neutral	Emotional	Neutral	Emotional	Neutral
Sleep Parameters	*M (SD)*	*M (SD)*	*M (SD)*	*M (SD)*	*M (SD)*	*M (SD)*
SWS%	27.69 (1.61)	25.31 (1.30)	27.62 (8.59)	26.34 (8.02)	28.43 (8.98)	25.16 (7.35)
REM%	18.67 (0.65)	19.17 (0.71)	18.58 (3.93)	20.08 (4.59)	18.87 (3.52)	17.89 (1.89)
Light sleep%	45.11 (1.50)	46.77 (1.52)	44.82 (8.40)	45.19 (7.98)	45.34 (7.67)	48.05 (9.14)
TST (min)	486.37 (6.44)	486.7 (5.06)	482.36 (46.24)	485.67 (28.54)	500.39 (22.58)	490.31 (27.51)
Sleep latency (min)	16.45 (3.34)	11.87 (2.55)	19.53 (21.22)	11.12 (15.35)	10.64 (11.56)	12.77 (11.72)
REM latency (min)	111.92 (8.19)	110.33 (5.24)	118.86 (48.48)	118.53 (29.08)	116 (53.61)	102.15 (27.59)
SWS latency (min)	18.97 (3.06)	16.5 (2.70)	20.75 (18.79)	21.08 (17.49)	15.68 (12.88)	10.32 (4.99)
N Awakenings	20.47 (1.57)	18.86 (1.53)	19.39 (7.36)	18.05 (7.86)	21.86 (10.66)	19.93 (9.82)
N REM periods	4.97 (0.19)	4.9 (0.14)	4.89 (0.83)	4.78 (0.09)	5.07 (1.33)	5.00 (0.58)
WASO (min)	34.34 (3.93)	32.24 (21.66)	36.7 (25.32)	31.64 (22.40)	31.08 (16.34)	33.08 (21.48)
REM fragmentation	0.35 (0.15)	0.34 (0.16)	0.36 (0.16)	0.37 (0.19)	0.34 (0.12)	0.3 (0.09)
Sleep efficiency (%)	90.87 (1.00)	91.77 (0.09)	90.0 (6.66)	91.94 (5.32)	92.79 (3.02)	91.85 (4.34)

All: all subjects; LSQR: low sleep quality responders; HSQR: high sleep quality responders;

SWS: Slow Wave Sleep; TST: Total Sleep Time; WASO: wake after sleep onset.

**Table 3:** Sleep architectural parameters in the emotional and neutral condition for the first and second night half.

	ALL (N = 32)		LSQR (N = 18)		HSQR (N = 14)	
Condition	Emotional	Neutral	Emotional	Neutral	Emotional	Neutral
Sleep Parameters	*M (SD)*	*M (SD)*	*M (SD)*	*M (SD)*	*M (SD)*	*M (SD)*
*First night half*						
SWS%	39.50 (11.46)	37.73 (9.55)	40.15 (12.50)	38.82 (9.77)	39.90 (11.00)	36.52 (9.12)
REM%	11.69 (4.96)	10.36 (3.47)	11.47 (4.82)	10.03 (3.90)	11.87 (5.15)	11.00 (2.82)
Light sleep%	41.65 (11.09)	44.40 (10.45)	41.04 (12.49)	43.33 (10.26)	41.42 (9.72)	45.52 (10.60)
*Second night half*						
SWS%	17.33 (8.47)	14.78 (7.33)	16.51 (8.15)	14.63 (7.17)	17.51 (9.44)	16.18 (8.77)
REM%	25.58 (4.98)	27.96 (6.20)	26.08 (5.86)	29.91 (6.72)	25.79 (4.81)	25.25 (4.32)
Light sleep%	48.40 (17.70)	48.23 (9.02)	48.52 (6.84)	46.97 (8.13)	48.64 (8.84)	50.75 (10.20)

All: all subjects; LSQR: low sleep quality responders; HSQR: high sleep quality responders.

**Figure 1:**
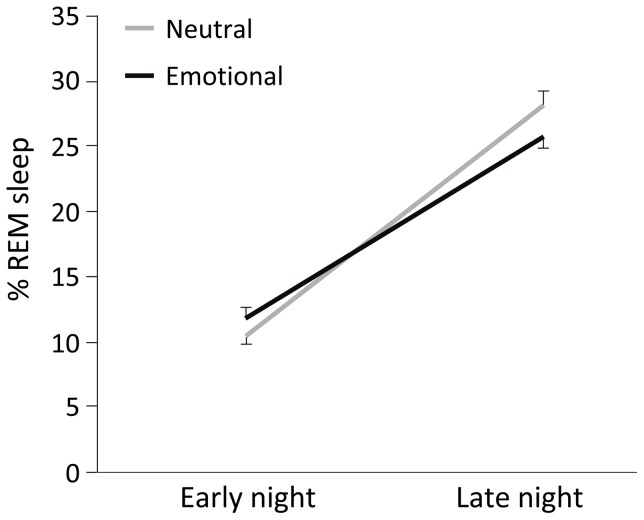
REM sleep percentages in the first and second night half in the complete sample. The natural increase in REM sleep percentage (means and SEM) from first to second half of the night was reduced after the emotional film (black line) as compared to the neutral film (grey line).

Results showed a significant increase of SWS% in the emotional film condition (main effect film: *F*(1,29) = 4.20, *p* = 0.05). Furthermore, the emotional film altered the distribution of REM sleep over the night (interaction effect of Film and Night half (*F*(1,29) = 4.62, *p* = 0.04). Indeed, the proportion of REM sleep normally increases over the night, such that most REM sleep occurs in the second half of the night. Following the emotional film this normal increase appeared to be reduced (Figure 1). Accordingly, there was a trend-level decrease in REM% in the second half of the night for the emotional versus the neutral condition (post hoc *t*(30) = −1.83, *p* = .077).

**Figure 2:**
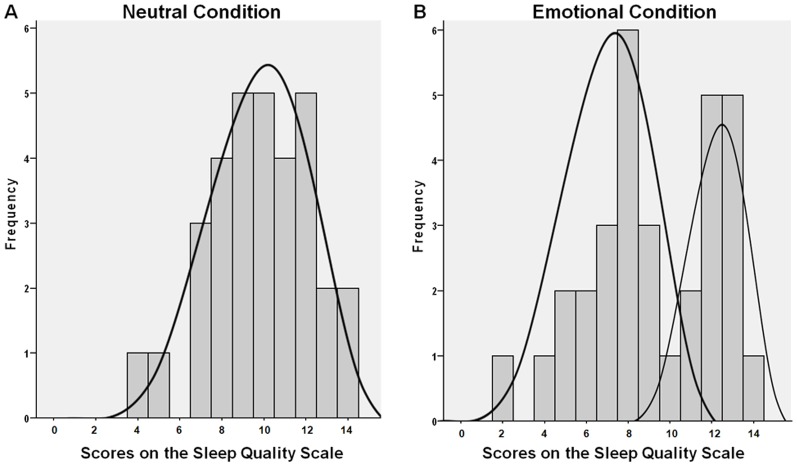
Frequency distribution of sleep quality scores in the neutral (A) and emotional (B) condition. The bimodal form of the distribution in panel B suggests the existence of a group with low sleep quality and a group with high sleep quality after the emotional film.

Statistical comparison of subjective sleep quality after viewing the emotional or neutral film fragment indicated no difference between these conditions (*t*(31) = −0.40, *p* = 0.70). However,

the distribution of sleep quality scale scores in the emotional condition appeared distinctly bimodal (Figure 2b); no such bimodality was observed in the distribution for the neutral condition (Figure 2a), nor in any other test variable in this study. Bimodality in emotional condition sleep quality scores was further checked, comparing the data to a unimodal and a bimodal distribution in MATLAB (Mathworks, inc., 'gmdistribution.fit' function). This confirmed that the bimodal distribution was the best fitting model, receiving the lowest Akaike Information Criterion (unimodal = 166.37, bimodal = 162.02) [47].

These findings suggest there may be individual differences in people's sleep response to emotional distress, with one type of subjects responding adaptively (slightly increased sleep quality), another in a maladaptive manner (reduced sleep quality). Even though we did not set out to investigate such individual differences we deemed it important to explore the sleep and emotional characteristics of these groups and assess each group's sleep architectural response to emotional distress.

### Individual differences in the sleep response to emotional distress?

**Figure 3:**
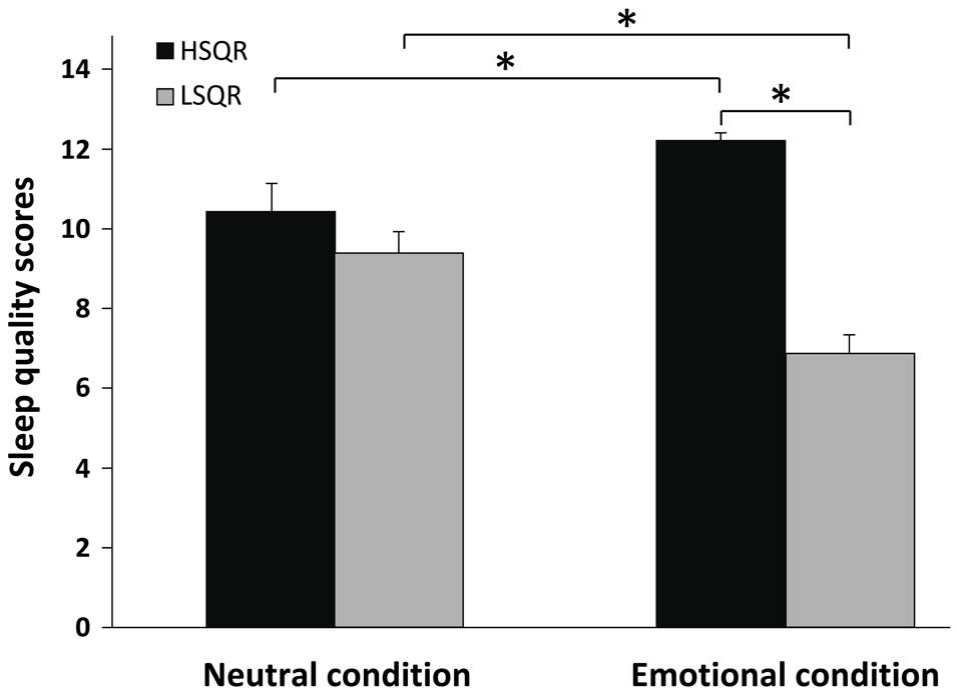
Sleep quality scores in HSQR (high sleep quality responders) and LSQR (low sleep quality responders). Means and SEM values of the sleep quality scores in the HSQR (black bar) and the LSQR (white bar) group are shown. The sleep quality of the 2 groups differed after the emotional film, but not after the neutral film. The sleep quality in the LSQR group decreased in the emotional night as compared with the neutral night, whereas the HSQR group showed a slightly increased sleep quality.

Based on the emotional sleep quality bimodal distribution fitting, subjects were divided into 18 ‘low sleep quality responders’ (LSQR: sleep quality scores≤9, 14F/4M) and 14 ‘high sleep quality responders’ (HSQR: sleep quality scores≥10, 9F/5M). Sleep quality scores in these two groups, after watching the neutral and emotional film, are given in Figure 3. These data were analyzed through ANOVA with factors Film (neutral, emotional) and Sleep quality type (HSQR, LSQR). We found a main effect of Sleep quality type (F(1,30) = 37.56; *p*≤*0.0009*) and an interaction effect of Film condition and Sleep quality type (F(1,30) = 17.72; *p*≤*0.0009*). Posthoc t-tests showed that LSQR had significantly lower subjective sleep quality after watching the emotional film in comparison to the neutral film (*t*(17) = −3.43, *p* = 0.003). Conversely, HSQR had slightly but significantly higher subjective sleep quality in the emotional compared to the neutral condition (*t*(13) = 2.65, *p* = 0.02). While HSQR had higher subjective sleep quality than LSQR on the emotional film night (*t*(30) = −9.38, *p*<0.001), scores were similar on the neutral film night (*t*(30) = −0.95, *p* = 0.35). Thus, the two groups differ in their sleep response to negative emotional experiences, rather than sleep quality in general.

We then investigated sleep architecture in the two groups (Tables 2 and 3). Ideally, this would have been addressed though ANOVA with factors Film (neutral, emotional), Sleep quality type (HSQR, LSQR) and Night-half (1^st^ half, 2^nd^ half). However, given considerable sleep architectural differences between individuals, such analysis has limited power to find differences between the relatively small and uneven-numbered sleep quality groups. To still gain some insight into potential sleep differences between HSQR and LSQR, the effects of Film and Sleep quality type were assessed in separate analyses.

Sleep architectural differences between the two groups were most apparent in the control condition. Indeed, the latency to SWS in LSQR was approximately twice as long as in HSQR (*t*(20.5) = 2.48, *p* = 0.022), while the REM increase over the night was considerably steeper in LSQR (298%) than in HSQR (233%; F(1,29) 5.73, p = 0.023). Accordingly, REM% in the second half of the night was significantly higher in LSQR (29.9 min) than in HSQR (25.2 min; *t*(28.7) = 2.35, *p* = 0.026). This was also the case in the emotional condition, but to a lesser, non-significant extent. Thus, the two groups differ in baseline sleep characteristics, with HSQR showing faster SWS onset and LSQR showing more prominent late night REM sleep.

**Figure 4:**
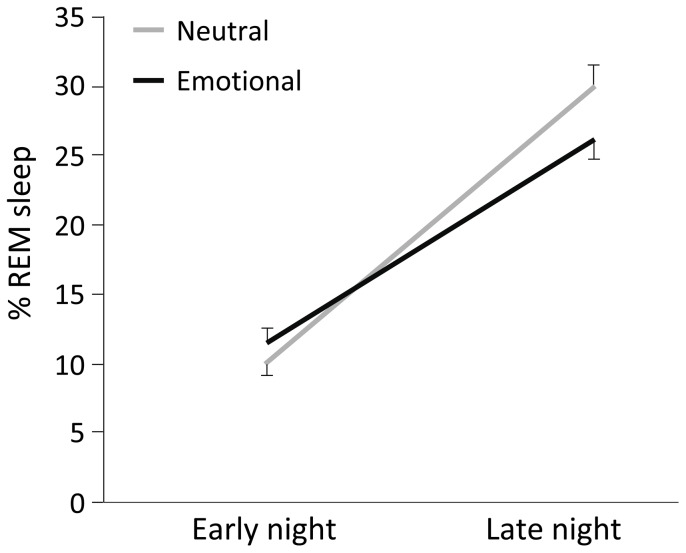
REM sleep percentages in first and second night half in LSQR in the two conditions. The LSQR group showed a reduced REM sleep percentage (means and SEM) in the second night half of the emotional night (black line) compared to the corresponding night half of the neutral night (grey line).

Next, we analyzed the effects of emotion on sleep architecture, for HSQR and LSQR separately. This showed that HSQR significantly increased SWS% from the neutral to the emotional condition (*t*(12) = 2.19, *p* = 0.048), while LSQR did not (*p*>0.4). On the other hand, LSQR appeared to be responsible for the flattened REM sleep distribution over the night in the emotional condition (interaction of Film and Night-half: *F*(1,17) = 6.45, *p* = 0.021; Figure 4). Indeed, this group significantly lowered REM% in the second night half, in the emotional compared to the neutral condition (post-hoc T-test: *t*(17) = −2.19, *p* = 0.043). This effect was not present in HSQR (*p*>0.6). LSQR also displayed a trend towards lowered sleep efficiency on the emotional film night (*t*(17) = −1.77, *p* = 0.094).

Finally, HSQR and LSQR subpopulations were compared with regard to emotional parameters. Interestingly, LSQR displayed a stronger emotional response (LSQR/HSQR*100) to the distressing film than HSQR, as reflected in the depression (182%, *z* = 2.0, *p* = 0.045), tension (148%, *z* = 2.03, *p* = 0.043) and global mood scores (149%, *t* = 2.595, *p* = 0.014).

These findings suggest that HSQR and LSQR present with subtle baseline sleep differences and respond differently to a presleep emotional stressor. HSQR show a more moderate emotional response to begin with and present with increased sleep quality and SWS% during subsequent sleep. On the other hand LSQR show a stronger emotional response to the distressing stimulus and respond with lowered sleep quality, flattening of the REM sleep increase over the night and a trend toward lowered sleep efficiency [48]. Notably, SWS and sleep efficiency are the main physiological correlates of subjective sleep quality [48].

### Correlations between emotional distress and ensuing sleep alterations

To further investigate the influence of emotional distress on sleep architecture, correlations were calculated between emotional responses to the distressing film (indexed by POMS and global mood scores) and sleep response variables (value in the emotional condition–value in the control condition) in the ensuing night. The latter variables reflect the alterations in sleep architecture induced by the emotional stimulus. These correlations, calculated both for the sample as a whole and for HSQR and LSQR separately, are given in Table 4.

**Table 4:** Correlations between stressor-induced emotional responses and sleep architectural alterations in the ensuing night.

Mood parameter	Sleep parameter	*r*	*p*
**All (N = 32)**			
Tension	Sleep efficiency	−0.44	0.012
Tension	SWS latency	0.42	0.016
Tension	N awakenings 1^st^ night half	0.41	0.019
Global mood	N REM periods	0.39	0.026
Global mood	Sleep efficiency	−0.39	0.029
Depression	Sleep latency	0.37	0.030
Global mood	SWS% 2^nd^ night half	0.36	0.045
**LSQR (N = 18)**			
Anger	SWS latency	0.54	0.021
Anger	N awakenings	0.50	0.034
Tension	N awakenings	0.48	0.046
Global mood	N REM periods	0.44	0.065
Tension	SWS latency	0.44	0.070
Tension	Sleep efficiency	−0.44	0.070
**HSQR (N = 14)**			
Depression	SWS% 2^nd^ night half	0.75	0.002*
Depression	SWS%	0.67	0.009
Depression	N REM periods	0.58	0.029
Tension	SWS latency	0.57	0.034

All: all subjects; LSQR: low sleep quality responders; HSQR: high sleep quality responders.

* Indicates the correlation is statistically significant after correction for multiple comparisons.

**Figure 5:**
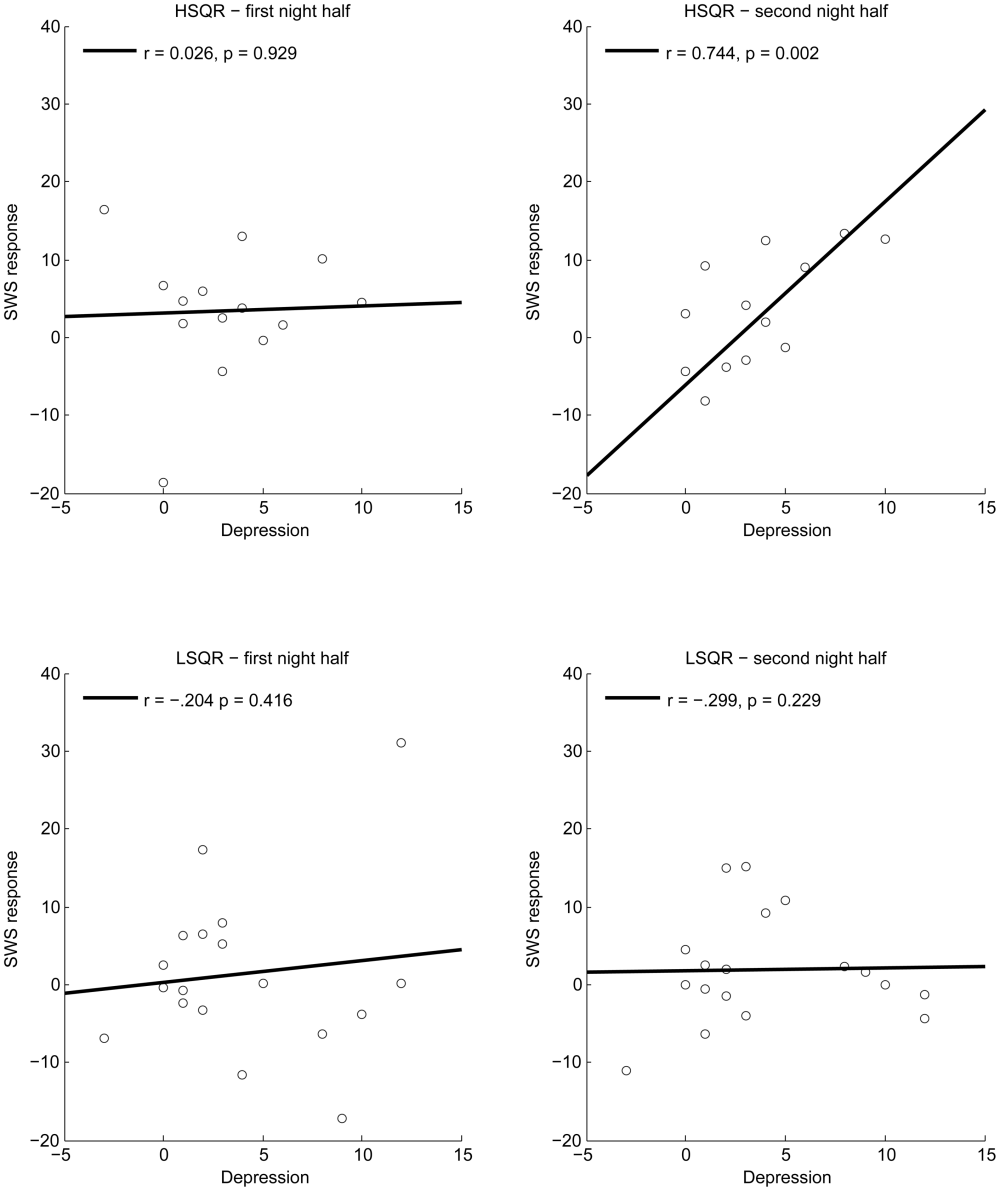
Correlations between depression scores and SWS% in the two night halves of the emotional night. HSQR (high sleep quality responders; upper panels) showed a very high correlation between induced depression in the emotional condition and SWS% increase in the second half of the ensuing night. In LSQR (low sleep quality responders; lower panels) no such correlation was observed.

In line with the comparative statistics, the correlation analyses show a positive relation between emotional distress and SWS% increase, especially in the second night half. The relation is modest in the sample as a whole, but very strong in HSQR alone (Figure 5). Notably, HSQR also showed the largest mean increase in SWS% following emotional distress. Interestingly, the analyses revealed a moderate positive relation between emotional impact and the number of REM sleep episodes during the night. This relation was observed in the sample as a whole, in HSQR and, at trend level, in LSQR. Finally, whole-sample correlations indicate mild to moderate tendencies towards sleep deterioration with increasing emotional impact, as reflected in longer sleep latency and latency to SWS, an increased number of awakenings and lowered sleep efficiency. The correlations with delayed SWS onset are significant in both groups. The other correlations are more apparent in LSQR, though some are then reduced to trend-level significance. Thus, the disrupting effects of emotional distress on sleep continuity may be contributed preferentially by LSQR.

Of the above correlations, the ones between emotional distress and SWS increase in HSQR are, by far, the strongest and also survive when α is corrected for multiple comparisons.

### Correlations between sleep alterations and emotional attenuation

The previous analyses indicate that acute emotional distress has subtle, but consistent, effects on oscillatory brain dynamics during sleep. We next explored whether the observed alterations in SWS and REM sleep patterning were related to the extent of emotional dissipation over sleep. To this purpose SWS and REM sleep responses (value in the emotional condition-value in the control condition) were correlated with cross-sleep emotional attenuation, expressed as the emotional response to the film minus the response to the cues. The analyses were controlled for size of the pre-sleep emotional response, which may influence the extent of emotional attenuation. Since this requires parametric statistics (partial correlations), emotion was indexed only by global mood scores, which were normally distributed. We found a moderate, positive relation between SWS% increase in the first half of the night and emotional attenuation (*r* = 0.5, *p* = 0.006). A more extensive analysis, considering all sleep parameters and including separate analyses for the HSQR and LSQR, is reported in supplementary Table S1 in File S1 and Figure S1 in File S1.

### Correlations between baseline sleep and emotional attenuation

While the previous section focused on sleep *responses* to distress, emotional coping characteristics could also be related to sleep *traits*. To explore this possibility, sleep architecture variables on the control night were correlated with cross-sleep emotional attenuation in the emotional condition (response to the film-response to the cues). We, again, focus on SWS and REM sleep, while more comprehensive analyses are reported online (Table S2 in File S1). Findings indicated a strong positive relation between baseline SWS% and emotional attenuation (whole night: *r* = 0.63, *p* = 0.006; first night half: *r* = 0.56, *p* = 0.007). Thus, a high SWS%, be it a baseline characteristic or a response to emotional distress, appears to favor emotional dissipation over sleep.

## Discussion

Our results show that an induced, emotionally distressing experience alters the electrophysiological patterning of subsequent sleep in healthy young adults. These alterations consist of a slight increase in the proportion of SWS and a flattening of the normal REM sleep increase over the course of the night. Importantly, two subpopulations can be discerned, based on a bimodal distribution of subjective sleep quality following the emotional stressor: one subpopulation shows a moderate emotional response to the distressing stimulus and responds with elevated SWS% and increased subjective sleep quality during the subsequent night (HSQR); the other shows a stronger emotional response to the distressing stimulus and responds with flattening of the REM sleep increase over the night and lowered sleep quality (LSQR). Interestingly, the two subpopulations also differ during control sleep: HSQR display a shorter latency to SWS, which may be seen as an adaptive trait, and lower REM% during the second night half compared to LSQR. Correlation analyses support the above and additionally show an increased number of REM periods with emotional distress. They, moreover, indicate a modest relation between emotional distress and sleep deterioration (reflected in increased sleep latency and latency to SWS, lowered sleep efficiency and increased numbers of awakenings), which seems to be contributed preferentially by LSQR.

As indicated in the introduction, previous studies reporting effects of acute psychosocial stressors on ensuing sleep architecture are few and present with methodological limitations. However, a more extensive and coherent body of evidence regarding these effects is available in rodents. Such studies have demonstrated a sharp increase in sleep EEG slow-wave activity (SWA; the EEG power density between 0.5 and 4 Hz) following social defeat stress [49], [50], [51], which dissipates over the course of approximately 12 hours. SWA and SWS being strongly related in humans [52], these findings are in line with our own.

The combined findings suggest that sleep debt and subsequent non-REM sleep intensity depend not only on the duration of prior wakefulness, as outlined in classic models of sleep regulation [53], but also on stressors experienced during the waking period. More specifically, acute emotional stressors may accelerate the build-up of SWS debt. As such, increases in SWS following emotional distress may reflect a compensatory response to effects of the stressor. The functional relevance of this response may relate to the ‘rest and restore’ function classically ascribed to SWS (e.g. in view of increased metabolic demand associated with emotion and arousal), but may also accommodate an increased need for information reprocessing during sleep. The latter view is fed by several studies implicating SWS in the ‘replay’ and consolidation of memory representations [24], [54], [55].

The notion of SWS increase as an adaptive response to emotional distress is supported by our finding that such increases predict cross-sleep emotional attenuation; especially when SWS increase occurs in the first part of the night, reinforcing normal sleep architecture. Interestingly, a very strong relation was also observed between baseline SWS% and emotional attenuation, in particular, again, for SWS in the first half of the night. This suggests that favorable SWS traits may protect against persistence of emotional distress. On the other hand, and perhaps not surprisingly, increased sleep disruptions (awakenings) after emotional distress were related to poor emotional dissipation over sleep (see correlation analyses in supplementary Table S1 in File S1).

Our other main finding regards a flattening of the REM sleep distribution over the night following emotional impact. A previous study [56] reports a somewhat similar observation, namely a flattening of the normal increase of REM density over sleep, following exposure to an acute psychological stressor. Furthermore, studies on sleep in depressed patients also show a flattened distribution of REM sleep over the night, accompanied by reduced REM latency and increased early night REM sleep [57]. Notably, in our study the stressor induced a marginally significant increase of REM% in the first 8^th^ segment of sleep (distressing film: Mean 1.18 min, SD 2.98; neutral film: Mean 0.10 min, SD 0.45; *t* = 1.97, *p* = 0.058) and a trend-level shortening of REM latency with increasing emotional impact in LSQR (*r* = −.45, *p* = 0.062). This pattern in healthy subjects may represent a mild form of what is seen in depression. It might, in this context, be considered that tendencies towards increased SWS, as observed in our sample, may counteract tendencies towards early night REM increase [58]. In comparison, major depression is associated with a reduction of SWS [57], which might allow a more pronounced manifestation of REM shift responses.

Another REM sleep related finding regards the positive correlation between emotional distress and the number of REM periods in subsequent sleep. Given that total REM sleep percentage does not notably increase with emotional distress, this finding, again, points to altered REM sleep patterning following emotional impact. One might speculate that such repatterning occurs consequent to altered pressure on the different sleep stages induced by emotional impact. More specifically, the REM shifts towards sleep onset may relate to an increased urgency for REM-related reprocessing of distressing event memories. Such notions are generally in line with other studies implicating REM sleep and dreaming in reprocessing of distressing information [38], [41], [59], [60], [61], [62], [63]. It should be noted however, that we did not find support for a relation between emotion-induced REM alterations and overnight emotional dissipation. This might reflect that REM sleep repatterning is not consistently related to emotional dissipation, at least not on the short term.

The above findings bring to mind observations of REM fragmentation in association to PTSD [18]. Of particular interest, one previous study reports on polysomnographic sleep measures in subjects within a month of life-threatening trauma and injury. The group that went on to develop PTSD symptoms was distinguished by a higher number of REM sleep periods and shorter average duration of continuous REM with respect to the group that did not develop PTSD. Follow-up PTSD severity was correlated positively with the number of REM sleep periods and negatively with the average duration of continuous REM sleep [64]. This suggests that increases in REM period number, and maybe REM fragmentation or repatterning in general, do not constitute a (short-term) adaptive response to emotional distress but, rather, may predict persistence of emotional distress and present a risk factor for PTSD.

Two other studies support this general notion: a recent study found that overnight arousal adaptation to aversive pictures increased with the extent of REM sleep deprivation, even after controlling for sleep efficiency and awakening times [65]. In another study [66], habituation to aversive pictures over a nap was larger than over an equally long wake period. However, occurrence of REM during the nap was negatively associated with arousal habituation (skin conductance response), while SWS correlated positively with habituation of the frowning (corrugator supercilii) response.

The above studies, at first glance, counteract the notion that REM sleep serves a cathartic role in emotional coping. However, this is not necessarily the case. For one, most findings implicating REM sleep in reprocessing of stressful information [38], [41], [59], [60], [61], [62], [63] or in emotional catharsis [41], [67], regard dream-related parameters, including dream content, dream emotions and REM density. It is currently not clear how such findings relate to those regarding REM sleep occurrence and repatterning. There is some evidence that REM sleep and dreaming do not have fully overlapping functional relevance in relation to emotional processing [65].

Finally, our discovery of subpopulations with differential sleep and emotional characteristics merits consideration. While to some extent preliminary, the findings suggest a coupling of certain emotion and sleep traits into distinct emotional sleep types. This means some people may be more able to cope with emotionally distressing experience than others, depending, to notable extent, on sleep traits.

Our combined findings have implications for affective disorders, such as PTSD and depression. Indeed, the close reciprocal relation between sleep and emotional processing in our study supports the notion that sleep problems may negatively affect emotional coping and contribute to the etiology of affective disorder. Moreover, the finding of emotional sleep types suggests that some persons might be more prone to the development of such disorders than others and that risk factors, possibly in the form of sleep-physiological markers, might be defined.

For further considerations pertaining to this study see the supplementary material File S1.

## Conclusions

In conclusion, we show that an emotionally distressing experience prior to sleep induces alterations in sleep architecture, involving tendencies towards mild sleep deterioration, but also a proportional increase of SWS and a flattening of the REM sleep distribution over the night. While sleep deterioration is negatively associated to emotional attenuation over sleep, a high SWS proportion in early sleep, whether it occurs as a trait or in response to the stressor, relates positively to such attenuation. Favorable SWS traits may thus protect subjects from persistent emotional distress, while distress-related SWS increase may form part of a compensatory response to the stressor. The REM sleep response to emotional distress bears partial similarity to REM abnormalities in depression and PTSD, but its potential functional relevance is currently unclear. We tentatively suggest such alterations in REM patterning may be related to relatively pronounced emotional distress and cross-sleep persistence rather than dissipation of the emotional state.

Importantly, we observed the existence of two subpopulations that differ in baseline sleep characteristics, emotional responsiveness and both subjective and electrophysiological sleep responses to emotional distress. The existence of these population subtypes warrants further research, assessing, for instance, whether the aptitude to cope with traumatic experience may be predicted based on sleep and emotional processing traits.

The combined results provide strong evidence for an intimate reciprocal relation between sleep physiology and emotional processing and highlight the importance of both REM sleep and SWS in this relation.

## Supporting Information

File S1 **Supporting information.** Supporting information including Figure S1 and Tables S1 and S2.
(DOC)
