# Supplementary material for: Sleeping Worries Away or Worrying Away Sleep? Physiological Evidence on Sleep-Emotion Interactions
Source: PLoS One. 2013 May 1;8(5):e62480. doi: 10.1371/journal.pone.0062480 (PMC3641038; doi:10.1371/journal.pone.0062480)
Supplement: File S1 — Supporting information. Supporting information including Figure S1 and Tables S1 and S2. (DOC) [file pone.0062480.s001.doc]

**Supplement to « Sleeping worries away or worrying away sleep? Physiological evidence on sleep-emotion interactions »**

**L.M. Talamini, L.F. Bringmann, M. de Boer & W.F. Hofman**

In this supplement we present additional results and discuss some limitations and considerations pertaining to the study described in the main text.

**Comparing cross-sleep emotional attenuation between HSQR and LSQR**

Since HSQR and LSQR differ with respect to certain emotional processing and sleep characteristics, these subpopulations might also differ with regard to emotional attenuation over sleep. Analyses on this point are only possible to a limited extent, as comparison of cross-sleep emotional attenuation (emotional response to distressing film – emotional response to stills) between the two groups requires controlling for the size of the pre-sleep emotional response, which may influence the extent of emotional attenuation. Since this requires parametric statistics (ANCOVA), emotion can be indexed only by global mood scores, which were normally distributed. At least on this mood parameter, there was no significant difference between groups (*F*(1,29) = 0.38, *p* = 0.54).

**Correlation analyses**

Our main paper presents a limited set of correlation analyses regarding the way in which sleep traits and sleep responses to emotional distress relate to emotional attenuation. More extensive correlation analyses, considering all emotion and physiological sleep parameters and including separate analyses for the whole sample, HSQR alone and LSQR alone, are presented here. The only sleep measure we did not consider is light sleep, in view of its known strong negative correlation with SWS.

For individual statistical tests α was set at 0.05. However, in view of multiple correlation tests, we also report which correlations are significant considering a family wise error rate of 0.05. In defining families of correlation tests, interdependency was assumed among the 4 emotion scales, and among certain sleep variables that are interdependent by definition (i.e. in view of the way they are calculated). This is the case for the proportional sleep stage measures, and also for sleep latency (light sleep was not considered), number of awakenings and sleep efficiency. Individual test values were corrected with the Bonferroni-Holm procedure (Holm, 1979).

Please note that the correlations reported in the main text are also included here for the sake of completeness.

*Correlations between sleep alterations and emotional attenuation*

The following correlations explore how emotion-induced sleep changes in sleep physiology are related to cross-sleep emotional attenuation (Table S1). Emotion-induced sleep alterations, both for the whole night and the two night halves separately, were expressed as the value in the emotional condition minus the value in the control condition. These ‘sleep response variables’ were correlated to cross-sleep emotional attenuation, expressed as the emotional response to the film minus the response to the cues. The correlation analyses were controlled for size of the pre-sleep emotional response, which may influence the extent of emotional attenuation. Since this requires parametric statistics (partial correlations), emotion was indexed only by global mood scores, which were normally distributed.

The sample as a whole shows a moderate, positive relation between SWS increase and emotional attenuation, especially when the increase is in the first half of the night, following the normal temporal distribution of SWS (significance maintained after controlling for multiple comparisons). This relation is particularly strong in LSQR. Results, furthermore, indicate a negative relation between sleep disturbances and emotional attenuation. Indeed, in the whole sample and for HSQR and LSQR separately, increased awakenings correlate negatively with mood attenuation over sleep.

**Table S1**. Correlations between sleep responses and cross-sleep emotional attenuation.

| **Mood parameter** | | **Sleep parameter** | ***r*** | ***p*** |
| --- | --- | --- | --- | --- |
| **All (N=32)** | | | | |
| Global mood attenuation | SWS 1^st^ night half | | 0.50 | 0.004* |
| Global mood attenuation | SWS | | 0.38 | 0.030 |
| Global mood attenuation | N awakenings | | -0.39 | 0.034 |
| Global mood attenuation | N awakenings 1^st^ night half | | -0.37 | 0.043 |
| **LSQR (N=18)** | | | | |
| Global mood attenuation | SWS 1^st^ night half | | 0.60 | 0.010 |
| Global mood attenuation | SWS | | 0.60 | 0.011 |
| Global mood attenuation | N awakenings 1^st^ night half | | -0.53 | 0.028 |
| **HSQR (N=14)** | | | | |
| Global mood attenuation | N awakenings 2^nd^ night half | | -0.58 | 0.050 |

All: all subjects; LSQR: low sleep quality responders; HSQR: high sleep quality responders

* Indicates that the correlation is statistically significant after correction for multiple comparisons

**Table S2.** Correlations between cross-sleep attenuation of stressor-induced emotional responses and baseline sleep parameters.

| **Mood parameter** | **Sleep parameter** | ***r*** | ***p*** |
| --- | --- | --- | --- |
| **All (N=32)** | | | |
| Depression attenuation | SWS | 0.63 | 0.000* |
| Depression attenuation | SWS 1^st^ night half | 0.56 | 0.001* |
| Anger attenuation | SWS | 0.41 | 0.020 |
| Depression attenuation | SWS 2^nd^ night half | 0.40 | 0.023 |
| **LSQR (N=18)** | | | |
| Depression attenuation | SWS | 0.55 | 0.018 |
| Depression attenuation | SWS 1^st^ night half | 0.51 | 0.032 |
| Depression attenuation | SWS 2^nd^ night half | 0.49 | 0.039 |
| Tension attenuation | REM | -0.46 | 0.054 |
| **HSQR (N=14)** | | | |
| Depression attenuation | SWS | 0.79 | 0.001* |
| Depression attenuation | SWS 1^st^ night half | 0.64 | 0.015 |
| Global mood attenuation | SWS 2^nd^ night half | 0.63 | 0.016 |
| Global mood attenuation | SWS | 0.59 | 0.033 |

All: all subjects; LSQR: low sleep quality responders; HSQR: high sleep quality responders

* Indicates the correlation is statistically significant after correction for multiple comparisons

*Correlations between baseline sleep and emotional attenuation*

As mentioned in the main text, emotional coping characteristics could also be related to sleep *traits*. To explore this possibility, sleep architecture variables on the control night were correlated with overnight emotional attenuation in the emotional condition. Again, this was done for the whole sample and for HSQR and LSQR separately (Table S2).

Findings point to a very strong positive relation between baseline SWS% and emotional attenuation, especially for SWS% in the first half of the night. These correlations occur in both subpopulations, but are particularly pronounced in HSQR (Figure S1). In addition, LSQR showed a marginally significant negative correlation between baseline REM% and tension attenuation.

Several of the correlations between baseline SWS and emotional attenuation maintain statistical significance after the correction for multiple comparisons.


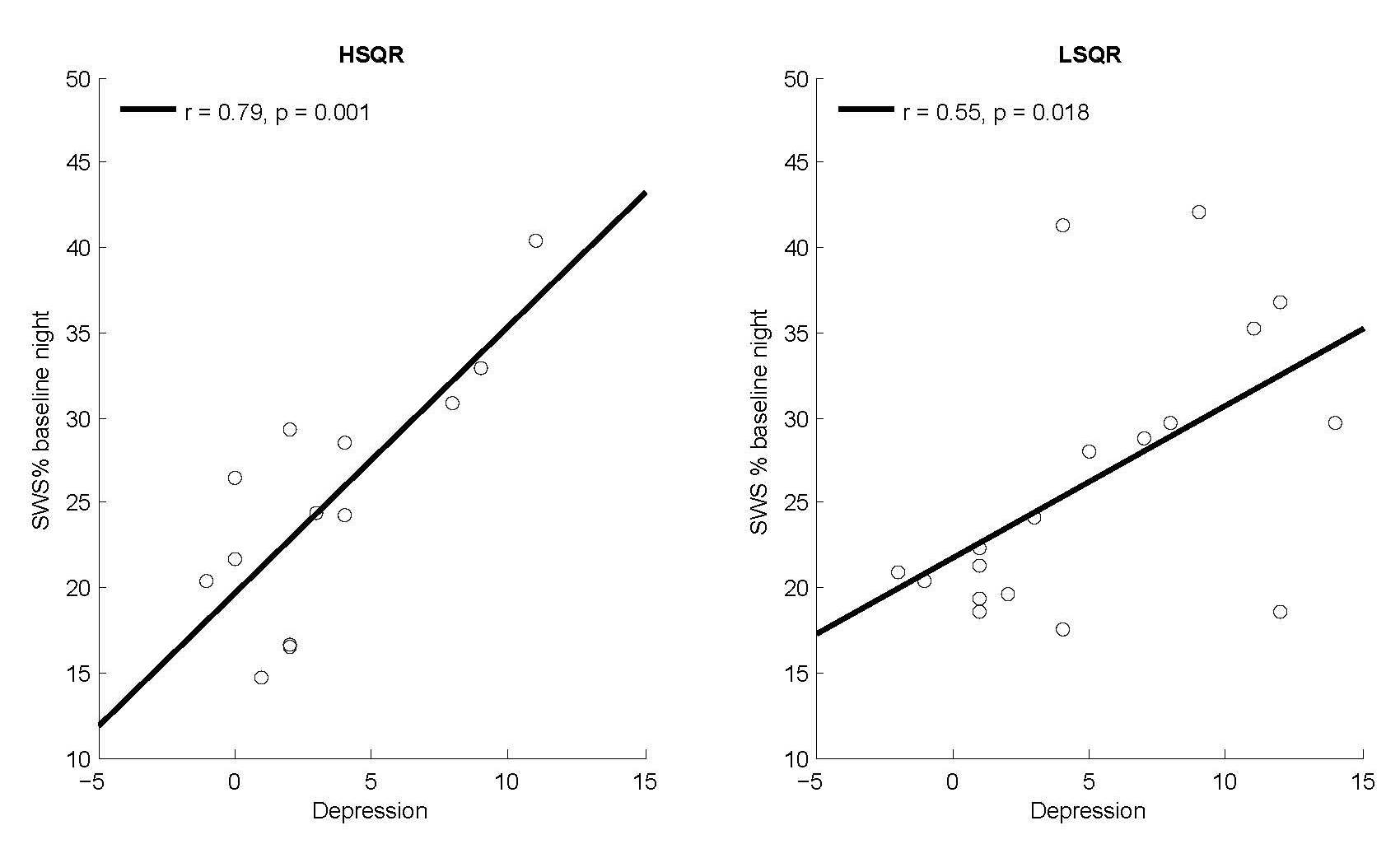


**Figure S1. Correlations between baseline SWS% and overnight depression attenuation in the emotional condition for HSQR and LSQR.** A higher SWS% in the control condition is related to more overnight attenuation in depression scores after watching the distressing film for both the high sleep quality responders group (HSQR) and the low sleep quality responders group (LSQR). The correlations are more pronounced in the HSQR group.

*Discussion of correlation analyses*

The correlation analyses are briefly discussed in the Discussion section of the main text. A more in depth discussion, especially regarding the differential findings in HSQR and LSQR is reported here.

As also mentioned in the main discussion, the correlation analyses support and extend the main findings, indicating a moderate positive relation between emotional distress prior to sleep and increased SWS% in the sample as a whole. This relation is inexistent in the LSQR, but very strong in HSQR, suggesting that the latter subjects may be mainly responsible for the correlation in the whole sample. There also appears to be a modest relation between emotional distress and sleep deterioration in the whole sample, reflected in increased sleep latency and latency to SWS, lowered sleep efficiency and increased numbers of awakenings. The negative effects on sleep continuity may be contributed preferentially by LSQR. Finally, the number of REM periods is shown to go up with emotional distress.

The emotion-induced SWS increase in HSQR is in line with this group’s slight increase in subjective sleep quality after emotional distress, while reduced sleep continuity in LSQR would be in line with this group’s reduced subjective sleep quality. Indeed, the strongest predictors of the sleep quality index are SWS and variables reflecting sleep continuity efficiency (Keklund and Ǻkerstedt, 1997).

Exploring a possible relation between sleep physiology and emotional catharsis, we show that SWS increases predict strong emotional attenuation; especially when they occur in the first part of the night, in line with normal sleep architecture. There is also a very strong, positive relation between baseline SWS% and emotional attenuation, especially, again, for SWS in the first part of the night. On the other hand, an increase in sleep disruptions (awakenings) after emotional distress is related to poor emotional attenuation over sleep, both in LSQR and in HSQR.

While the relation with baseline SWS is more pronounced in HSQR, the relation with SWS responses is stronger in LSQR. This may reflect that the short-latency, baseline SWS pattern in HSQR favors emotional attenuation, but leaves little room for further stressor-induced enhancement in early sleep. Accordingly, emotional distress and SWS increase in this group correlate most strongly in the second half of sleep. Apparently, such late night SWS increases are not significantly related to emotional attenuation. On the other hand, the baseline SWS distribution in LSQR may leave more room for early night, stressor induced SWS increases, which do correlate with emotional attenuation.

**Limitations and considerations**

Some limitations of the study should be addressed. First, due to the fact that we did not set out to investigate the existence of subgroups of sleep responders, the subsamples of HSQR and LSQR are somewhat limited in size. Replication with larger samples might strengthen some of the findings regarding these subgroups and the differences between them. Second, it should be noted that sleep architectural differences between HSQR and LSQR in the control condition (neutral film) might involve differential responses to sleeping in a novel environment (the sleep lab). The extent to which such differences are also reflected in habitual sleep remains to be investigated. Thirdly, the correlations between sleep parameters and emotional attenuation were controlled for size of the pre-sleep emotional response and, thus, only concerned Gaussian variables. While this enhances the interpretability of results, relevant relations might have been missed.

Finally, it might be considered that the broad spectrum of human emotions may have a varied influence on sleep. The emotion induction procedure in our own study tended to induce a rather depressed state, which was also the emotional element that correlated most strongly with SWS parameters. In comparison, certain other mood induction procedures instill a nervous or apprehensive state in subjects (e.g. regarding some personal challenge or evaluation on the subsequent day). Such studies tend to find more prominent sleep disruption than our own, which may be related to the aroused state, with worry and rumination about post-sleep events (De Koninck and Koulack, 1975; Vandekerckhove et al., 2011). The potentially different effects of varying emotional states on sleep and the recovery process merit further investigation in future studies.

**References**

De Koninck JM, Koulack D (1975) Dream content and adaptation to a stressful situation. *J Abnorm Psychol* 84(3):250-60.

Holm S (1979) A simple sequential rejective multiple test procedure. *Scandinavian Journal of Statistics* 6:65–70.

Keklund G, Ǻkerstedt T (1997) Objective components of individual differences in subjective sleep quality. *J Sleep Res*. 6(4):217-220.

Vandekerckhove M, Weiss R, Schotte C, Exadaktylos V, Haex B, Verbraecken J, Cluydts R (2011) The role of presleep negative emotion in sleep physiology. *Psychophysiology* 48:1738–1744.
